# Phytogenic Feed Additives as a Sustainable Alternative to Antibiotics: Enhancing Growth and Disease Resistance in Nile Tilapia (*Oreochromis niloticus*) †

**DOI:** 10.3390/ani15030380

**Published:** 2025-01-28

**Authors:** Christina Gruber, Vladimira Ocelova, Jutta C. Kesselring, Silvia Wein

**Affiliations:** Animal Nutrition & Health R&D Center, DSM-Firmenich, 3430 Tulln, Austria; vladimira.ocelova@dsm-firmenich.com (V.O.); jutta.kesselring@dsm-firmenich.com (J.C.K.); silvia.wein@dsm-firmenich.com (S.W.)

**Keywords:** phytogenic feed additive, Nile Tilapia, growth performance, immunity, resistance, *Streptococcus agalactiae*

## Abstract

Pathogens and challenging conditions in fish farming are common problems that cause mortality and reduce growth performance. Phytogenic feed additives can support the immunity of fish and thereby improve performance, production, and disease resistance. Two experiments with Nile Tilapia aimed to evaluate the effects of a matrix-encapsulated phytogenic additive (Digestarom^®^ P.E.P. MGE) in different feed formulations, which vary in the marine meal inclusion level (5% and 12.8%), on growth performance and resistance against *Streptococcus agalactiae*. Fish were stocked in a recirculating aquaculture tank system per experiment and were fed with diets with or without the phytogenic supplementation for 8 weeks (six replicates per group). After an intraperitoneal injection with a sterilized saline solution and bath immersion with *S. agalactiae* (5 × 10^6^ CFU/mL), mortality of the fish was recorded for 20 days. Independent of the diet formulation, the supplementation with the phytogenic feed additive significantly improved the fish production; feed conversion ratio; immune response (mainly lactic acid dehydrogenase, differential blood cell counts, and the lysozyme activity in blood); and fish survival after the *S. agalactiae* challenge. Thus, phytogenic feed additives are promising strategies to improve Nile tilapia production by enhancing fish growth performance, health, and protection against *S. agalactiae* infections.

## Simple Summary

Disease outbreaks due to bacterial pathogens are among the biggest challenges in the aquaculture industry, resulting in mortality, impaired growth performance, and, subsequently, loss of profit. Antibiotic treatments are commonly used to control bacterial pathogens. However, since the development of antibiotic resistance is a concern, innovative antibiotic-free solutions are needed to replace their use. The usage of formulated functional aquafeeds incorporating additives, such as phytogenic compounds, represents a key strategy for enhancing animal performance and resilience against diseases. The results of two experiments with Tilapia demonstrate that phytogenic feed additives are promising strategies to improve fish production and provide increased protection against bacterial infection, independent of the diet formulation.

## 1. Introduction

The global demand for animal protein for human consumption is steadily increasing due to continuous population growth. According to the Food and Agriculture Organization (FAO), approximately 17% of all animal protein consumed globally is currently provided by fish [1]. Aquaculture has grown consistently over the last decades, reaching about 51% of the global fish production in 2022 [1]. To meet the global demand for animal protein, aquaculture is expected to further increase substantially in the coming years. Innovative strategies need to be developed to increase aquaculture production, sustainability, resilience, efficiency, and profitability. For example, production efficiency and animal health, especially in intensive and super-intensive production systems, can be largely achieved by high-quality seed and genetic improvement programs, specifically selective breeding and vaccinations [1]. Formulated functional aquafeeds that include feed additives can further enhance resilience to disease, as well as enable more sustainable aquaculture practices, especially with regards to the efficient use of raw materials such as fish meal [2].

Phytogenic feed additives (PFAs) are plant-derived products and can improve the growth and health of animals in livestock and aquaculture production [3,4,5,6]. Their positive effects on appetite stimulation, growth promotion, antimicrobial, antioxidant, anti-inflammatory, and immunomodulatory properties are caused by bioactive substances, such as alkaloids, flavonoids, glycosides, phenolics, saponins, tannins, terpenoids, steroids, and essential oils [7,8].

PFAs are classified based on their botanical origin, processing methods, and composition and can comprise of different herbal products, including spices, aromatic plants, oleoresins, and essential oils. Plant essential oils are a major group of PFAs that usually include a mixture of hydrocarbons (terpenes and sesquiterpenes); oxygenated compounds (alcohol, aldehydes, esters, and ketones); and a small percentage of nonvolatile residues (e.g., paraffin and wax) [9]. They can have similar benefits as antibiotic growth promoters, such as antimicrobial, antioxidant, anti-inflammatory, and immunomodulatory activities, while, at the same time, often improving the growth and feed efficiency of aquatic organisms [9,10]. Recent studies demonstrate that plant essential oils can improve the growth performance and health of aquatic organisms [7,8,9,10,11,12,13].

Furthermore, plant essential oils can improve feed palatability, gut functions, and antioxidant responses in terrestrial animals [14,15]. Recent studies have also demonstrated that PFAs can compensate for the negative consequences of replacing fishmeal with plant-based protein by improving nutrient retention, growth performance, and disease resistance in aquatic organisms [12,16,17,18].

However, the functional mechanisms supporting the benefits of PFAs can be species-specific [10]. Furthermore, the effect of the phytogenic feed additive may also depend on the feed formulation, e.g., the fishmeal inclusion level in a nutrient-sparing context [12,16,17,18]. Therefore, the aim of this study was to investigate the effects of plant essential oils on the growth performance and health of Nile Tilapia, *Oreochromis niloticus*, fed diets with different marine meal levels (5% and 12.83%). Tilapia is the second-most common group of farmed fish after carps [1]. Tilapia is a popular species in aquaculture due to its fast growth and robustness towards a wide range of environmental conditions and diet compositions. In early life, their diet is omnivorous, and therefore, they are fed with aquafeeds containing lower levels of fishmeal and higher plant-derived proteins [19]. Although tilapias are rather resistant to stress and diseases, they are susceptible to bacterial pathogens, including *Streptococcus* spp., *Flavobacterium columnare*, *Aeromonas hydrophila,* and *Edwardsiella tarda*, and parasitic infections, e.g., *Ichthyophthirius multifiliis* and *Trichodina* spp. [20,21]. Especially, *Streptococcus* spp. can cause severe health problems in farmed tilapia that result in economic losses of up to 250 million USD globally [20]. One of the most problematic *Streptococcus* species to the tilapia industry worldwide is *S. agalactiae*, which causes clinical symptoms from decreased feed intake to unilateral or bilateral exophthalmos, eye hemorrhages, corneal opacity, distended abdomen, curvature of the spinal cord, stiffness, erratic swimming, and bleeding at the base of the fins [21,22,23]. Commonly, antibiotic treatments are used to control *Streptococcus* infections [21]. However, since the development of antibiotic resistance is a concern, innovative antibiotic-free solutions are needed to replace antibiotic use. The present study examined the effect of a commercially available matrix-encapsulated phytogenic feed additive that is based on essential oils of oregano, thyme, and citrus (Digestarom^®^ P.E.P. MGE, Biomin GmbH, Herzogenburg, Austria) on the growth performance, immune response, and resistance against *Streptococcus agalactiae* of Nile tilapia fed different diets that, among other things, vary in the marine meal level (5% and 12.83%).

## 2. Materials and Methods

Two separate experiments testing different feed formulations, hereafter defined as 5% (low) or 12.83% (high) marine meal diets (Table 1), were performed at the Aquaculture Center for Applied Nutrition (Vietnam). Sex-reversed male Nile tilapias (*O. niloticus*) were obtained from a local hatchery and housed in a 2000-L quarantine tank under standard recirculating water conditions for 14 days before the onset of each experiment. During this acclimation period, all fish were fed the commercial diet that the fish received in the hatchery three times per day until apparent satiety. Those diets had a similar crude protein (32%) and different fat levels (5 or 8%).

Both experiments took place in 12 tanks of a recirculating aquaculture system (RAS), evaluating the effects of 0.2 g/kg Digestarom^®^ P.E.P. MGE (matrix-encapsulated phytogenic additive based on essential oils of oregano, thyme, and citrus, Biomin GmbH, Herzogenburg, Austria). The dietary inclusion level of the phytogenic product represents the minimum recommended dosage by the manufacturer and is known to positively affect the growth performance and immunity of aquatic animals, e.g., [12]. Two experimental groups (Table 1) with six replicates each were randomly assigned to the tanks without (C) or with supplementation with the phytogenic feed additive (PFA) in the 5% (low) and 12.83% (high) marine meal diets (Table 1).

For the first experiment using a low marine meal diet, 840 Nile tilapia (mean body weight ± standard deviation: 10.63 ± 0.01 g) were randomly placed in twelve 560-L tanks (70 fish/tank) of dimensions 100 cm × 100 cm and a 56 cm water level. For the second experiment using a higher marine meal diet, 600 Nile tilapia of the fast-growing GenoMar Gain strain (mean body weight ± standard deviation: 25.20 ± 0.02 g) were also randomly allocated in twelve 270-L tanks (50 fish/tank) of dimensions 70 cm × 70 cm and 55.11 cm water level. The higher marine meal diet was chosen to better meet the nutrient requirements of the larger fast-growing fish strain.

The diets were mixed according to the formulation (Table 1). The feed ingredients were ground and mixed to produce a basal mash diet. The feed additive Digestarom^®^ P.E.P. MGE was then added to this basal diet at a dose of 0.2 g/kg and mixed using a 10 L Hobart-type mixer. Distilled water (40% *v*/*w*) was added to the dry mash so that the diets could be pelleted using a meat grinder (maximal temperature 55 ± 5 °C), dried in an airflow oven at 60 °C for 48 h, broken to the adjusted pellet size, and sieved to remove any dust. The dry pellets were kept in feed containers and stored in the air-conditioned storage room at 2 °C. Feed samples were taken to analyze the nutrient composition (Table 1).

During the 56-day feeding trial, fish were fed to near satiety four times per day. Feeding behavior, feed intake, and mortality were recorded for each tank daily to estimate the amount of feed provided in the subsequent meals. The fish were weighed after feeding the experimental diets for 56 days, and the feed consumption during the trial period was quantified. Fish were weighed after 24 h of starvation and under anesthesia (2-phenoxyethanol 0.2 mL/L provided via immersion in water). The feed conversion ratio (FCR) was calculated as the proportion of the total feed consumption to the fish weight gain per tank. Specific growth rate (SGR) was calculated according to the following formular:

$$SGR=\frac{ln\left(\mathrm{final}\;\mathrm{mean}\;\mathrm{fish}\;\mathrm{weight}\right)-\ln\left(\mathrm{initial}\;\mathrm{mean}\;\mathrm{fish}\;\mathrm{weight}\right)}{time\;(days)}*100$$

After the 56-day feeding trial, a subsequent challenge experiment with *Streptococcus agalactiae* was conducted. A total of 20 fish from each replicate were randomly selected for the challenge trial and placed in reciprocal 80-L tanks (55 cm length, 40 cm wide, and water level 36 cm in height) in the challenge room for the subsequent challenge trial. In total, 12 independent challenge tanks with a small biofilter each were used. The animals of the different groups were housed in different tanks and continued to receive the experimental diet (2 groups with 6 tanks/replicates each).

The animals were acclimatized for 2 days in the challenge tanks to adapt to the new conditions and ensure that no mortality occurred after the transfer. The challenge included two steps: a primary treatment and a bacteria immersion challenge.

For the primary challenge treatment (mock injection), fish received an intraperitoneal injection (IP) with 0.1 mL of a sterilized saline solution (9 g NaCl/L distilled water).

Four days later, the fish were infected with *S. agalactiae* (purchased from the strain collection of the Fish Pathology Lab, Faculty of Fisheries, Nong Lam University, Ho Chi Minh City, Vietnam, which isolated the strain from diseased fish). The procedure included the transfer of individual fish from the challenge trial to an immersion bath containing 10 L of water, followed by the addition of 100 mL bacterial broth to achieve the target bacterial concentration dosage of 2.12 × 10^7^ CFU/mL. The fish were challenged for 1 h and were then placed back in the challenge tank, where they were held until the end of the trial. During the post-challenge period, fish were fed at 2% of body weight [28] divided into two daily meals. The previously developed challenge model reached 50% mortality 14 days after the challenge (unpublished data). Water in the tanks was replaced twice daily prior to feeding to maintain normal water quality. Water temperature was 29. 5 °C ± 1.0 °C, dissolved oxygen 5.41 ppm ± 0.5 ppm, pH 7.45 ± 0.5, total ammonia < 0.25 ppm, and nitrites < 0.5 ppm (mean ± SD). The nitrogen parameters were TAN < 0.25 ppm and nitrites < 1 ppm. The water quality parameters were checked and recorded regularly and maintained at these conditions for the studies. For the measurements, a handheld oxygen meter (Oxi 3210 Set 1 DO Meter WTW 2BA201, WTW, Weilheim, Germany), a handheld pH meter with pH sensor InLab Expert Pro (S20-K SevenEasy™ pH, Mettler Toledo, Columbus, OH, USA) and API^®^ test kits (API Inc., Chalfont, PA, USA) were used.

Mortality was recorded daily for 20 days, starting from the primary challenge and through the 16 days that followed the bacterial challenge. Fish were observed twice daily during the challenge trial. Moribund and dead fish were removed. To confirm the infection and determine the cause of death, deceased fish were sent to the Nong Lam University lab for examination via real-time PCR.

In each experiment, animals were randomly selected per tank at the end of the performance trial and seven days after the bacterial challenge. In the trial testing the 5% marine meal diet, two fish per tank were randomly chosen before and after the challenge for blood sampling to analyze hematology and plasma lysozyme, and another two fish per tank were sampled for plasma enzyme analysis (12 fish per group, timepoint, and analysis). After anesthesia, those animals were sacrificed for sampling.

In the trial testing the 12.83% marine meal diet, five fish per tank were randomly chosen for blood sampling to analyze hematology and plasma lysozyme, and another five fish per tank were sampled for plasma enzyme analysis (30 fish per group and analysis). The same animals were sampled before and after the challenge. The sampled fish were individually marked with a color thread in the tail for identification.

Blood was collected to determine the (i) blood cell count (erythrocytes, leucocytes, hemoglobin, hematocrit, lymphocyte, monocyte, and neutrophil cells); (ii) lysozyme activity; and (iii) activity of plasma enzymes (lactic acid dehydrogenase (LDH), aspartate aminotransferase (AST), alanine aminotransferase (ALT), and alkaline phosphatase (ALP)). Following sedation, blood (500 µL) was withdrawn from the caudal vein with a 1.0-mL syringe coated with EDTA 10% solution equipped with a 26-gauge hypodermic needle. The blood samples were kept in 2 mL EDTA-K2 (Sequestrene) plastic tubes for further analysis. The hematocrit was immediately measured in a glass capillary tube after centrifugation at 10,000 rpm for 5 min in a microhematocrit centrifuge. The hemoglobin levels were colorimetrically quantified after reaction to cyanmethemoglobin. Red and white blood cells were determined in a hemocytometer after dilution. Leukocyte differentiation was quantified by differential counting under the microscope. Blood smears were stained with May–Grunwald Giemsa Wright.

To assess lysozyme activity, 1 mL of blood was centrifuged at 10,000× *g* at 4 °C for 10 min. The serum was then separated and stored at −20 °C for the quantification of lysozyme activity with a turbidimetric assay (Sigma-Aldrich Co. LLC, St. Louis, MO, USA). The turbidimetric assay was carried out according to [29] with some modifications. Briefly, 190 µL of a *Micrococcus lysodeikticus* suspension (0.20 mg per mL 0.04 M sodium phosphate buffer, pH = 6.2) was mixed with varying amounts of lysozyme source and put in replicate wells of a 96-well microplate. The reduction in absorbance was measured at 540 nm after 1 min and 5 min. A unit of lysozyme activity is defined as the amount of plasma causing a decrease in absorbance of 0.001 per minute.

To analyze the LDH, AST, ALT, and ALP levels, 1 mL of blood per fish was collected from 12 randomly selected fish per experimental group. Blood was collected using a 1.0 mL syringe equipped with a 26-gauge hypodermic needle and a 2 mL serum tube. The samples were analyzed using an ADVIA 1800 Clinical Chemistry System (Siemens, Erlangen, Germany). All the analyses were performed at the Nong Lam University labs and the Medic Medical Center, HCMC, Vietnam.

At the end of the trial, all remaining fish were sacrificed, disinfected, and discarded. To sacrifice the animals, they were first anesthetized (2-phenoxyethanol (Sigma-Aldrich Co. LLC, St. Louis, MO, USA) 0.2 mL/L provided via immersion in water for 5 min [30,31]) and then transferred into ice water for 10 min. The sacrificed animals were stored in labeled plastic bags and kept at −18 °C.

All procedures were performed in compliance with relevant laws and institutional guidelines, and the appropriate institutional committee has approved them. All animal experiments comply with the ARRIVE guidelines and were carried out in accordance with the U.K. Animals (Scientific Procedures) Act, 1986 and associated guidelines, EU Directive 2010/63/EU for animal experiments, and the National Institutes of Health guide for the care and use of Laboratory animals (NIH Publications No. 8023, revised 1978). Sample sizes were determined using power calculations (G*Power, version 3.1). Researchers were unaware of the group allocation at all stages of the experiment and data analysis.

Boxplots were used to visually inspect the data distribution, variability, and outliers. The model assumptions (normality, homoscedasticity, and independence) were checked via the residual plots. No data were removed from the statistical analysis. Independent samples *t*-test (parametric), and Mann–Whitney *U* test (non-parametric) were used to test for differences between the treatments. Repeated measures ANOVA was used to compare the response in the hematological parameters across the groups and sampling timepoints before and after the challenge in the high marine meal diet experiment. Post hoc Tukey’s HSD test was used for multiple comparisons at the different timepoints. All statistics were done in the statistical program R (version 3.5) and JASP (Version 0.19.0). The significance level was defined as *p* < 0.05.

This article is a revised and expanded version of a paper entitled “Effect of a Phytogenic Feed Additive on the Growth Performance, Immune Response and Resistance against *Streptococcus agalactiae* of Nile Tilapia, *Oreochromis niloticus*”, which was presented at the 78th Conference of the Society of Nutrition Physiology in Göttingen, Germany, from the 5th to 7th of March 2024 [32].

## 3. Results

Compared to the control, the PFA supplementation of the 5% marine meal diet significantly improved the cumulative tank biomass after 8 weeks of feeding the diets (Table 2). This was paralleled by a significant decrease in FCR (−8.8%, Table 2, *p* < 0.05). Across groups using the 5% marine meal diet, similar survival rates and feed intake were observed (Table 2).

The PFA supplementation of the 12.83% marine meal diet significantly increased the mean final body weight, cumulative tank biomass, and SGR compared to the control group, and this was also accompanied by a significant decrease in FCR (−7.44%, Table 2, *p* < 0.05). Across groups, no differences in survival rate and feed intake were observed after eight weeks of being fed the 12.83% marine meal diet (*p* > 0.05).

Following the challenge with *S. agalactiae*, the PFA supplementation of the 5% marine meal diet resulted in lower mortality as compared to the control group (Table 2, *p* < 0.05). Similarly, the PFA supplementation of the 12.83% marine meal diet significantly reduced mortality of the tilapias after *S. agalactiae* as compared to the non-supplemented control group (*p* < 0.05, Table 2).

No mortality was observed from the primary challenge treatment, i.e., the IP injection with 0.1 mL of a sterilized saline solution, prior to the *S. agalactiae* immersion challenge in either experiment.

After being fed the 5% marine meal experimental diet for 57 days, similar levels of the hematological parameters were observed across the groups (*p* ≥ 0.098, Table 3a).

After the immersion challenge with *S. agalactiae*, ALT activities were significantly higher when the 5% marine meal diet was supplemented with the PFA compared to the non-supplemented control group (*p* = 0.022, Table 3b). Furthermore, the number of erythrocytes (*p* = 0.038), as well as the hemoglobin (*p* < 0.001) and hematocrit levels (*p* = 0.028), were higher when the 5% marine meal diet was supplemented with the PFA compared to the non-supplemented control group (Table 3b).

After being fed the 12.83% marine meal experimental diets for 57 days, LDH activities were significantly higher in the non-supplemented control as compared to the PFA group (*p* = 0.014, C: 406.67 ± 50.76 and PFA: 264.19 ± 57.71), whereas the lysozyme activity was significantly higher in the PFA group (*p* = 0.028, C: 485.83 ± 44.09 and PFA: 613.89 ± 35.61, mean ± standard error). At the respective sampling points, similar levels of the other immune parameters were observed across the groups (*p* ≥ 0.084).

**Figure 1:**
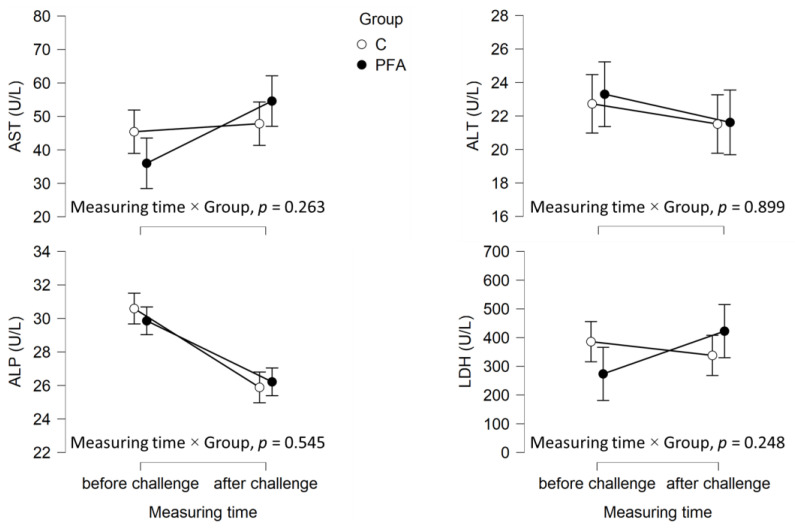
Mean ± standard error (SE) of aspartate aminotransferase (AST, U/L), alanine aminotransferase (ALT), alkaline phosphatase (ALP, U/L), and lactic acid dehydrogenase (LDH, U/L) of *Oreochromis niloticus* fed the high marine meal diet without (group = C) or with the phytogenic feed additive Digestarom^®^ PEP MGE at 0.2 g/kg feed (PFA) measured at the end of the performance trial (i.e., before challenge) and 7 days after the bacterial challenge in the same animals per group. The results of the interaction between measuring time and group from the repeated measure ANOVAs are provided.

**Figure 2:**
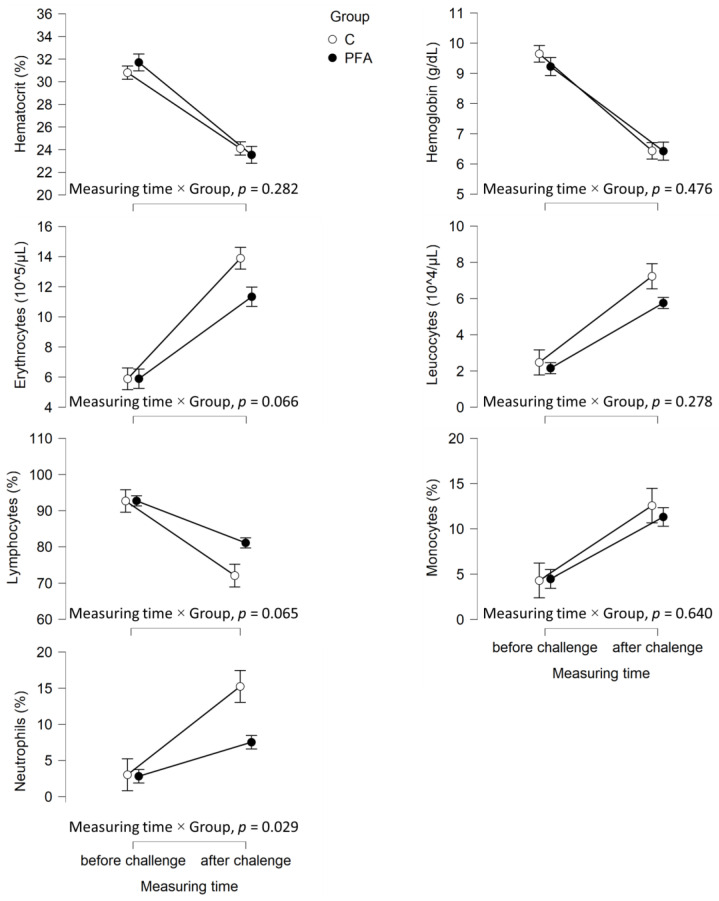
Mean ± standard error (SE) of the hematological parameters of *Oreochromis niloticus* fed the high (12.83%) marine meal diet without (group = C) or with the phytogenic feed additive Digestarom^®^ PEP MGE at 0.2 g/kg feed (PFA) measured at the end of the performance trial (i.e., before the challenge) and 7 days after the bacterial challenge in the same animals per group. The results of the interaction between measuring time and group from the repeated measure ANOVAs are provided.

**Figure 3:**
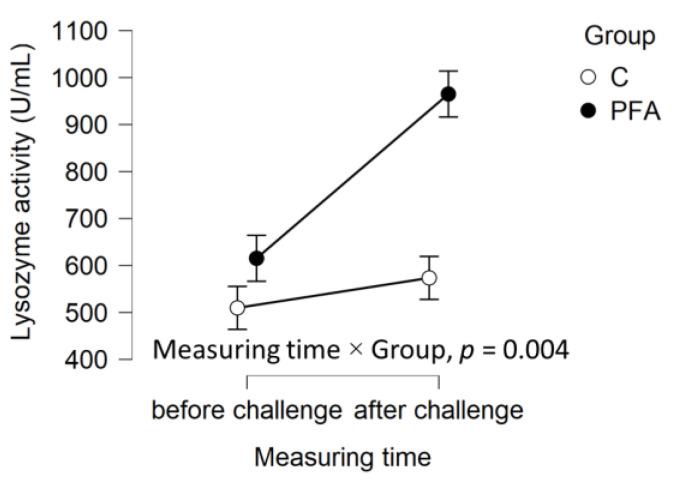
Mean ± standard error (SE) of lysozyme activity of *Oreochromis niloticus* fed the high (12.83%) marine meal diet without (group = C) or with the phytogenic feed additive Digestarom^®^ PEP MGE at 0.2 g/kg feed (PFA) measured at the end of the performance trial (i.e., before challenge) and 7 days after the bacterial challenge in the same animals per group. The results of the interaction between measuring time and group from the repeated measure ANOVAs are provided.

Out of the 30 animals per group, 23 fish from the control group and 24 fish from the PFA group were sampled a second time after the bacterial challenge (Figure 1, Figure 2 and Figure 3). When sampling the same animals again seven days after the bacterial challenge, significant interactions between measurement time and group for neutrophil levels (Figure 2), as well as lysozyme activity (Figure 3), were found. After the challenge, the mean neutrophil levels were significantly lower and lysozyme activity significantly higher in the PFA group than in the non-supplemented control group (Tukey’s post hoc analysis, *p* ≤ 0.007). At the respective sampling points, similar levels of all the other hematological parameters were observed across the groups (*p* > 0.05).

## 4. Discussion

To meet the increasing global demand for animal protein in the future, aquaculture requires innovative strategies to increase production, sustainability, resilience, efficiency, and profitability. The usage of formulated functional aquafeeds that include feed additives, such as plant essential oils, is one of those strategies. Phytogenic feed additives have been reported to compensate for the negative consequences of replacing fishmeal with plant-based protein by improving nutrient retention, growth performance, and disease resistance in aquatic organisms [12,16,17,18]. Various studies document the positive effect of PFAs on disease resistance ([8], challenge with *Edwardsiella ictaluri* [11], and challenge with *Aeromonas hydrophila* [33]). This can, in addition, help to reduce antibiotic and medical treatments.

The current experiments indicate that supplementing a diet with a blend of plant essential oils (Digestarom^®^ P.E.P. MGE) improved the growth performance and feed conversion, as well as disease resistance against *S. agalactiae*, independent of the used basal feed formulation. The overall mortality rate after *S. agalactiae* infection was approximately one-third lower in the supplemented group than in the group that received control feed with low or high marine meal levels in their diet. Our findings are in accordance with other studies that have demonstrated the positive effects of plant essential oils on growth performance, health, and disease resistance in various aquatic organisms [7,8,9,10,11,12,13,33].

Previous studies suggest that the mode of action of plant essential oils is related to the modulation of the gut bacterial composition, intestinal morphology, and immune and other physiological responses of the hosts [9]. Plant essential oils can provide anti-inflammatory and antioxidant activities, as well as influence the amount and type of secretions produced by the intestinal mucosa and alter the physical and chemical properties of the intestinal environment [9].

In this study, we examined hematological parameters that are generally used as indicators of condition and health, as well as the nutritional status of fish [34,35]. Although significant group differences were found in some of the measured parameters, especially after the challenge, all animals that were sampled at the respective sampling timepoints showed typical physiological age- and size-related hematological parameters. The enzymes measured in this study, including aspartate aminotransferase (AST), alanine aminotransferase (ALT), alkaline phosphatase (ALP), and lactate dehydrogenase (LDH), are within the physiological range typically observed in tilapia [36,37,38]. While ALT is primarily a liver enzyme, AST and ALP are found in various tissues, and LDH, in particular, is present in many tissues, including muscle, heart, liver, and kidneys. These enzymes provide valuable information about overall metabolic health [36,37,38].

In the experiment with the low marine meal diet, we found similar levels of the hematological parameters across the groups after feeding fish the experimental diets for 57 days. In the experiment with the high marine meal diet and higher number of fish sampled per group, we found lower mean LDH activities and higher lysozyme activity in the group supplemented with the phytogenic feed additive prior to the challenge. In a previous study with shrimp, we observed a decrease in LDH activity with supplementation of the same phytogenic feed additive [12], which might indicate reduced hepatic damages and infection levels [39]. The higher lysozyme activity in the phytogenic group is in accordance with other reported results in different fish species, indicating the immune-stimulating properties of phytogenic feed additives [40,41].

After the bacterial challenge in the experiment with the low marine meal diet, hematocrit, hemoglobin levels, and the number of erythrocytes were significantly lower in the control than in fish supplemented with the blend of plant essential oils. That could lead to a subsequent reduced oxygen supply to the tissue and impaired energetic performance, so the animals in the control group may have had an impaired health status and, hence, were more susceptible to disease [42]. In contrast, supplementation with the blend of plant essential oils seemed to have improved immune function, as can be clearly seen by the significantly improved survival rate after the bacterial challenge. Ergena et al. [43] described a similar reduction of hematocrit, hemoglobin levels, and number of erythrocytes after infection of Nile tilapia with *Aeromonas hydrophila*. Those results indicate that the infection impacted erythrocytes, both their total number and the percentage of erythrocytes in the blood volume, resulting in anemia. In addition to the anemic response, the decrease in hemoglobin after challenge can be explained by an increased rate of breakdown of red blood cells by pathogenic bacteria and/or the reduction in their production rate [43]. Compared to the control, they observed that the supplementation of the diet with ginger significantly increased the erythrocytes, hematocrit, and hemoglobin levels after the challenge, indicating stimulated hematopoiesis and a protective effect of the phytogenic due to antioxidant properties that protect against hemolysis by free radicals [43]. The present results are in accordance with the described beneficial effect of phytogenic supplementation. There might also be dietary effects on the hematological profile of the fishmeal-free diet. It is important to note that differences in the hematological levels may be influenced by interindividual differences or factors such as bacterial infection strain, water temperature, or dietary protein level [44]. Therefore, we increased the number of sampled animals and repeatedly sampled the same animals in the subsequent experiment with the high marine meal diet.

When sampling the same animals before and after the bacterial challenge in the high marine meal diet experiment, group differences in the response of neutrophil levels, as well as lysozyme activity, were found. Generally, the significant increase in both neutrophils and lysozyme activity indicates the immune defense response after the challenge. The observed differences between the groups in the two experiments might also be related to the different dietary compositions, stocking densities, and tilapia strain. However, the rise in lysozyme activity was significantly steeper in the group supplemented with the phytogenic feed additive than in the control, which follows previous observations [40,41]. Lysozyme is a cornerstone of innate immunity. In addition to its direct antimicrobial role, more recent evidence has shown that lysozyme modulates the host immune response to infection [45].

Neutrophils act as the first line of the non-specific innate immune response against pathogens and play an important role in the acute inflammatory response [46]. The percentage of neutrophils was significantly higher after bacterial infection than before, indicating a clear response to the bacterial infection. The increase in the percentage of neutrophils after the challenge was steeper in the control group, which indicates the positive effect of phytogenic supplementation stimulating fish antibodies against infections and returning them early to homeostasis [46]. Fish in the control group likely still rely on the activated non-specific immune response to fight the infection.

## 5. Conclusions

The present experiments using different diet formulations, which, among others, vary in the marine meal levels validate a nature-based solution for improving growth performance and disease resistance in Nile tilapia by supplementing the diets with a blend of essential oils. The improved disease resistance of tilapia following the supplementation with Digestarom^®^ P.E.P. MGE, independent of the diet, suggests that the PFAs can be successfully incorporated into diverse diets with no or low fishmeal inclusion rates to improve the health of the animals. Thus, including phytogenic feed additives into diets can be a promising strategy to improve Nile tilapia production by enhancing fish growth performance, and protection against *S. agalactiae* infections. Consequently, phytogenic feed additives can offer solutions to reduce the need for antibiotics and medical treatments.

## Figures and Tables

**Table 1:** Feed composition of the test diets without (control = C) or supplemented with the phytogenic feed additive (PFA, 0.2 g/kg Digestarom^®^ P.E.P. MGE).

Experiment	5% Marine Meal Diet	12.83% Marine Meal Diet
Ingredient	Inclusion Rate [%]	Inclusion Rate [%]
Whole wheat (13.5% protein)	31.20	17.30
Soybean meal (46% protein)	26.08	19.19
Rice bran	12.00	12.00
Meat and bone meal, 48% protein	8.49	12.00
Corn gluten	4.05	12.00
Feather meal	4.00	
Local fish meal 60% protein		7.33
Shrimp waste meal	3.00	3.00
Fish soluble, 37% protein	2.00	2.50
Hemoglobin powder—spray dried	2.00	
Wheat bran		7.00
Fish oil	1.00	1.34
Liquid lecithin	0.71	1.22
Soybean oil	1.00	1.33
Vitamin & Mineral Premix	0.25	0.25
MonoCaPhosphate	1.66	1.32
Limestone	1.00	1.00
Salt	0.50	0.50
Lysine HCL	0.30	0.16
Methionine	0.30	0.10
Choline Chloride 50%	0.10	0.10
Threonine	0.10	0.10
Taurine	0.10	0.10
Betaine	0.10	0.10
Vitamin C	0.06	0.06
**Analytical results**		
Moisture ([24], %)	12.18	5.65
Crude fiber ([25], %)	3.52	3.87
Crude fat ([26], %)	6.01	8.48
Protein ([27], %)	31.82	32.04
Carbohydrates (calculation, %)	41.00	41.85
Ash ([24], %)	9.00	11.98
Gross energy (calculation, kcal/kg)	3463.5	3329

**Table 2:** Growth performance and feed utilization of tilapia after 8 weeks of feeding a 5% (1) or 12.83% (2) marine meal diet without (C) or with (PFA) the supplementation of the diet with the phytogenic feed additive Digestarom^®^ PEP MGE 0.2 g/kg feed. Values are the means ± standard deviation.

Experiment	(1) 5% Marine Meal Diet (70 Fish/Tank)		(2) 12.83% Marine Meal Diet (50 Fish/Tank)	
	C	PFA	C	PFA
Survival (%) during 8 weeks of experimental feeding	92.86 ± 4.14	95.48 ± 1.67	95.00 ± 1.92	96.33 ± 0.75
Initial tank biomass (g)	744 ± 1	744 ± 0	1260 ± 1	1259 ± 1
Final tank biomass (g)	2464 ± 126 ^a^	2681 ± 177 ^b^	4828 ± 86 ^a^	5135 ± 47 ^b^
Initial body weight (g)	10.63 ± 0.01	10.63 ± 0.01	25.20 ± 0.02	25.19 ± 0.01
Final body weight (g)	37.93 ± 1.82	40.15 ± 3.31	101.67 ± 2.81 ^a^	106.61 ± 0.87 ^b^
Specific growth rate (%)	2.27 ± 0.09	2.37 ± 0.14	2.32 ± 0.03 ^a^	2.42 ± 0.02 ^b^
Feed intake (g/fish/day)	44.66 ± 1.59	44.28 ± 0.95	92.76 ± 2.02	91.46 ± 0.69
Feed conversion ratio	1.69 ± 0.09 ^a^	1.54 ± 0.13 ^b^	1.21± 0.03 ^a^	1.12 ± 0.01 ^b^
Mortality (%) after bacterial challenge	47.50 ± 5.24 ^a^	30.00 ± 3.16 ^b^	50 ± 12.65 ^a^	35 ± 5.48 ^b^

Superscript letters indicate significant differences (*p* < 0.05) per row of the specific experiment using the *t*-test or Mann–Whitney *U* test. C = diet without phytogenic supplementation and PFA = diet with supplemented phytogenic Digestarom^®^ P.E.P. MGE 0.2 g/kg feed.

**Table 3:** Mean ± standard error of the hematological parameters of *Oreochromis niloticus* fed a 5% marine meal diet without (control = C) or with the phytogenic feed additive Digestarom^®^ PEP MGE at 0.2 g/kg feed (PFA) (a) after feeding fish the experimental diets for 8 weeks and (b) after the bacterial challenge (*n* = 12).

Parameter	(a) After Feeding the Experimental Diets for 8 Weeks		(b) 7 Days After Bacterial Challenge	
	C	PFA	C	PFA
Aspartate aminotransferase (U/L)	57.96 ± 14.16	44.95 ± 7.18	26.45 ± 3.31	27.89 ± 2.74
Alanine aminotransferase (U/L)	20.97 ± 2.66	24.61 ± 3.70	19.93 ± 1.21 ^a^	25.72 ± 2.00 ^b^
Alkaline phosphatase (U/L)	34.06 ± 3.12	27.87 ± 1.76	23.71 ± 1.85	21.28 ± 3.31
Lactic acid dehydrogenase (U/L)	586.42 ± 147.70	338.10 ± 75.49	226.16 ± 40.66	256.94 ± 40.03
Erythrocytes—Total red blood cell (10^5^/mm^3^)	8.82 ± 0.38	8.92 ± 0.38	7.49 ± 0.36 ^a^	8.53 ± 0.30 ^b^
Leucocytes—Total white blood cell (10^4^/mm^3^)	3.96 ± 0.20	3.97 ± 0.24	3.98 ± 0.16	4.33 ± 0.21
Hematocrit (%)	32.71 ± 1.98	33.63 ± 1.88	17.63 ± 1.62 ^a^	22.67 ± 1.40 ^b^
Hemoglobin (g/dL)	11.73 ± 0.75	11.81 ± 0.64	7.05 ± 0.44 ^a^	9.07 ± 0.32 ^b^
Lymphocyte (%)	86.88 ± 1.85	88.38 ± 0.77	88.83 ± 1.11	90.00 ± 0.71
Neutrophil (%)	5.71 ± 1.03	5.58 ± 0.67	4.54 ± 0.51	4.67 ± 0.70
Monocyte (%)	7.42 ± 1.06	6.04 ± 0.48	6.04 ± 0.89	5.83 ± 0.62
Lysozyme activity (U/mL)	570.84 ± 87.33	731.94 ± 132.27	680.56 ± 110.05	587.50 ± 136.83

Superscript letters indicate significant differences (*p* < 0.05) per row of the specific experiment using the *t*-test or Mann–Whitney *U* test. C = diet without phytogenic supplementation and PFA = diet with supplemented phytogenic Digestarom^®^ P.E.P. MGE 0.2 g/kg feed.

## Data Availability

The data presented in this study are available on request from the corresponding author.
